# Relationship Between Severe Respiratory Depression and Codeine-Containing Antitussives in Children: A Nested Case-Control Study

**DOI:** 10.2188/jea.JE20180224

**Published:** 2020-03-05

**Authors:** Sachiko Ono, Yosuke Ono, Daisuke Koide, Hideo Yasunaga

**Affiliations:** 1 Department of Biostatistics & Bioinformatics, The University of Tokyo, Tokyo, Japan; 2 Department of General Medicine, National Defense Medical College, Saitama, Japan; 3 Department of Clinical Epidemiology and Health Economics, School of Public Health, The University of Tokyo, Tokyo, Japan

**Keywords:** codeine, antitussive, children, respiratory depression

## Abstract

**Background:**

Guidelines recommend against all codeine use in children for its common indications of analgesia and cough suppression because of uncertain benefits and potential risk of death. However, because of its rarity, the occurrence of severe respiratory depression associated with codeine-containing antitussives has been poorly investigated. The objective of this study was to investigate the association between codeine-containing antitussives and severe respiratory depression in children.

**Methods:**

We retrospectively identified Japanese children who were prescribed antitussives for respiratory diseases from a large Japanese administrative claims database (JMDC, Tokyo, Japan). We collected data on baseline characteristics including age, sex, and comorbidity. Each case was matched with four controls with the same sex and age in the same year from the same type of medical institution. We then examined the association between codeine-containing antitussives and subsequent severe respiratory depression using multivariable conditional logistic regression analysis.

**Results:**

Of 164,047 children, 18,210 (11.1%) were prescribed codeine-containing antitussives. Of the children who took codeine-containing drugs, seven experienced severe respiratory depression. After adjusting for confounding factors, there was no significant difference in the proportion of severe respiratory depression between children with and without codeine-containing antitussives (odds ratio 1.15; 95% confidence interval, 0.48–2.78).

**Conclusion:**

Occurrence of respiratory depression was very rare, and the association of codeine with respiratory depression was insignificant, even in a large sample of children in Japan.

## INTRODUCTION

Codeine is an opioid drug that has been used as a pain reliever and an antitussive. Children who are ultra-rapid metabolizers convert 5 to 30 times more codeine to morphine than do normal metabolizers,^1^^–^^3^ which can lead to fatal toxicity.^4^^–^^6^ There have been several reports of fatalities associated with standard doses of codeine among ultra-rapid metabolizers,^4^^–^^6^ although these fatal case reports have been limited to children who underwent tonsillectomy and/or adenoidectomy.^5^

For children, national and international guidelines recommend against all use of codeine for the common indications of analgesia and cough suppression because of the uncertain benefits of codeine^7^ and its potential risk of death.^8^^–^^11^ Nonetheless, people in both the United States and Japan can obtain over-the-counter drugs for children that contain codeine—mostly antitussive preparations—partly because the estimated population of ultra-rapid metabolizers is quite small.^5^

Epidemiological evidence of the risks associated with codeine-containing antitussives is scarce. Only one case report of codeine-containing antitussive intoxication in twins has been published.^12^ In Japan, following an international movement of guideline changes, the Ministry of Health, Labour and Welfare investigated the risk of codeine use in children under 18 years of age and reported that the incidence of severe respiratory depression was less than 1%.^13^ However, the study only included patients in seven academic hospitals. Consequently, the number of children was small (*n* = 408). Furthermore, generalizability of the study was limited because a wide variety of codeine-containing drugs are mainly prescribed for the common cold in primary care settings in Japan. Therefore, the risk of codeine-containing drugs among children in Japan remains uncertain.

The association between codeine-containing antitussives and severe respiratory depression has not been elucidated because of the rarity of the complication. Therefore, we investigated the incidence of severe respiratory depression and analyzed the association between codeine-containing antitussives and severe respiratory depression among Japanese children using a large administrative claim database.

## METHODS

### Data source

We conducted a nested case-control study using an administrative claim database, the JMDC, collected from April 2012 through December 2015. The JMDC is contracted with more than 60 insurers and collected data on annual health lifestyle disease screening records linked with health insurance claim data for approximately 1.5 million insured individuals in 2013. The JMDC provides researchers with de-identified data on health insurance beneficiaries and their families.^14^ The majority of insured individuals in the JMDC database are employees of Japanese companies. In the database, diagnoses are recorded based on International Classification of Diseases, 10th revision (ICD-10) codes. Drugs and procedures were recorded using European Pharmaceutical Market Research Association codes and Japanese medical procedure codes, respectively. Given the de-identified nature of the data, the requirement for informed consent was waived. The study was approved by the institutional review board of The University of Tokyo.

### Data extraction

We identified children younger than 18 years who were prescribed antitussives (European Pharmaceutical Market Research Association code: R05D) for respiratory disease (ICD-10 code: J) in the period from April 2012 through December 2015 and had at least 6 months of participation in the JMDC database before the prescription and at least 1 month of participation after the prescription. We set the beginning of the study period as April 2012 because the JMDC data included detailed prescription information, such as date of prescription, from this date onwards. Antitussives were categorized as with or without codeine (identified by the Japanese key word of “codeine”). We excluded children who were prescribed oxymetebanol, the only opioid other than codeine-containing drugs used as an antitussive in Japan. We also excluded children with diagnoses of malignancy and those who underwent tonsillectomy and/or adenoidectomy during the baseline period. We defined asthma/chronic obstructive pulmonary disease (COPD) by identifying prescriptions for anti-asthma and COPD products (European Pharmaceutical Market Research Association code: R03), including β2-agonists, xanthines, respiratory antihistamines, non-steroidal respiratory anti-inflammatory products, corticoids, phosphodiesterase 4 inhibitors, and anticholinergics, before the first antitussive prescription. We also identified diagnoses of malignant neoplasm (ICD-10 code: C), obstructive sleep apnea syndrome (OSAS) (G473), epilepsy (G40 and G41), liver disease (K7), and renal disease (K17–19) as potential confounders, based on previous studies. We used Japanese medical procedure codes to identify tonsillectomy and adenoidectomy before the first prescription of antitussives.

### Outcome

To define severe respiratory depression, we used a composite outcome indicating (i) medical conditions that required tracheal intubation or mechanical ventilation within a week after the prescription of antitussives or (ii) the combination of respiratory depression (ICD-10 code: J960 and R060) diagnosis and oxygen administration within a week after the prescription of antitussives.

### Data analysis

We fitted a linear regression model to analyze change in the prescription of codeine-containing antitussives among all antitussives over time, setting the proportion of codeine-containing drugs as the dependent variable and time (months) as an independent variable.

Children’s characteristics are presented as counts (proportion) or means (standard deviation). To compare variables between the codeine group and the other antitussives group, Student’s *t*-tests were used for continuous variables, and chi-square tests were used for categorical variables.

For the nested case-control study, each child with severe respiratory depression was matched with four control patients without severe respiratory depression. Control patients were prescribed antitussives at the same time point as the case patients but did not experience subsequent severe respiratory depression. Control patients with the same sex and age as the case were selected in the same year of antitussive prescription and from the same type of medical institutions.

Univariable analyses were conducted to examine the association between potential risk factors and severe respiratory depression. Multivariable conditional logistic regression was used to examine the association between codeine use and respiratory depression. Significant factors in the univariable analyses were included as independent variables. For the sensitivity analyses, we excluded children who were prescribed anti-asthma or COPD products during the baseline period and repeated the same analyses. All statistical analyses were two-tailed, and values of *P* < 0.05 were considered significant. All statistical computations were performed with Stata/MP V.14.2 (StataCorp, College Station, TX, USA).

## RESULTS

**Figure 1.**
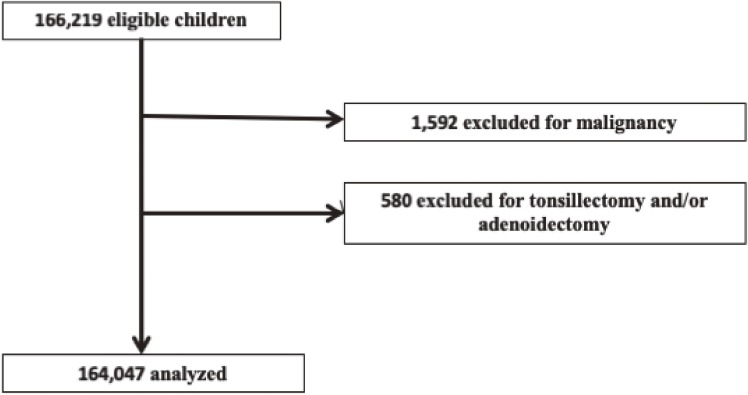
Patient selection

There were 166,219 children younger than 18 years who were prescribed codeine or other antitussive drugs for respiratory disease during the study period and who had at least 6 months of JMDC data participation before the prescription and at least 1 month of participation after the prescription. Of these children, 1,592 were excluded because of the presence of malignancy, and 580 were excluded because of tonsillectomy during the baseline period. None of the children were prescribed oxymetebanol during the study period. The remaining 164,047 children were divided into the codeine (*n* = 18,210) and other antitussive (*n* = 145,837) groups (Figure 1).

The proportion of codeine-containing drugs among antitussives declined over time during the study period (−0.004% per month; 95% confidence interval, −0.007 to −0.002).

Table 1 shows the patients’ characteristics. Approximately 98% of codeine-containing drugs were prescribed in primary care settings. Children in the codeine group were older and less likely to have contraindications for codeine, including asthma (defined by prescriptions of anti-asthma or COPD drugs), OSAS, or epilepsy.

**Table 1.** Characteristics of children prescribed antitussives for respiratory conditions

Factor	Other antitussives	Codeine	*P*
*N*	145,837	18,210	
Age, years, mean (SD)	7.3 (4.8)	10.3 (5.1)	<0.001
Female	69,573 (47.7%)	8,705 (47.8%)	0.8
Anti-asthma/COPD drugs	97,734 (67.0%)	10,160 (55.8%)	<0.001
Renal disease	306 (0.2%)	45 (0.2%)	0.30
Liver disease	7,771 (5.3%)	1,018 (5.6%)	0.14
COPD	25 (<1%)	6 (<1%)	0.14
OSAS	825 (0.6%)	82 (0.5%)	0.048
Epilepsy	2,633 (1.8%)	263 (1.4%)	<0.001
Medical institution			
Clinic	116,118 (79.6%)	17,868 (98.1%)	<0.001
Hospital	28,638 (19.6%)	337 (1.9%)	
Teaching hospital	1,081 (0.7%)	5 (<1%)	

COPD, chronic obstructive pulmonary disease; OSAS, obstructive sleep apnea syndrome; SD, standard deviation.

Numbers are presented as mean (standard deviation) for age and *n* (%) for categorical variables.

A total of 405 children experienced severe respiratory depression. Among the 18,210 children who were prescribed codeine-containing drugs, seven (approximately 3.8 per 10,000 children) experienced severe respiratory depression.

The case-control matching created 405 pairs of cases (*n* = 405) and controls (*n* = 1,620). Table 2 shows patient characteristics in the case-control-matched children with and without severe respiratory depression. The proportions prescribed codeine were not significantly different between the cases and the controls.

**Table 2.** Characteristics of case-control-matched children with and without severe respiratory depression

	Cases (with severe respiratory depression)	Controls (without severe respiratory depression)	*P*
*N*	405	1,620	
Age, years, mean (SD)	3.4 (3.3)	3.4 (3.3)	1.00
Female	170 (42.0%)	680 (42.0%)	1.00
Medical institution			1.00
Clinic	30 (7.4%)	120 (7.4%)	
Hospital	370 (91.4%)	1,480 (91.4%)	
Teaching hospital	5 (1.2%)	20 (1.2%)	
Codeine or other antitussives			0.79
Other antitussives	398 (98.3%)	1,595 (98.5%)	
Codeine	7 (1.7%)	25 (1.5%)	
Anti-asthma/COPD drugs	368 (90.9%)	1,308 (80.7%)	<0.001
Renal disease	2 (0.5%)	8 (0.5%)	1.00
Liver disease	40 (9.9%)	113 (7.0%)	0.048
OSAS	3 (0.7%)	12 (0.7%)	1.00
Epilepsy	22 (5.4%)	45 (2.8%)	0.008

COPD, chronic obstructive pulmonary disease; OSAS, obstructive sleep apnea syndrome; SD, standard deviation.

Numbers are presented as mean (standard deviation) for age and *n* (%) for categorical variables.

Although potential confounders, such as anti-asthma/COPD drug use and epilepsy, were significantly associated with severe respiratory depression, there was no significant association between codeine use and severe respiratory depression (odds ratio 1.15; 95% confidence interval, 0.48–2.78) (Table 3). The sensitivity analyses yielded similar results.

**Table 3.** Multivariable conditional logistic regression analysis for severe respiratory depression

	Odds ratio	95% confidence interval	*P*
Codeine or other antitussives			
Other antitussives	Ref.		
Codeine	1.15	0.48 to 2.78	0.76
Anti-asthma/COPD drug	2.52	1.73 to 3.67	<0.001
Epilepsy	1.80	1.05 to 3.10	0.033
Liver disease	1.37	0.92 to 2.04	0.12

COPD, chronic obstructive pulmonary disease.

## DISCUSSION

This was the first study to examine the incidence of severe respiratory depression after the use of codeine-containing antitussives in all clinical settings among children from the general population. We found non-significant differences in the incidence proportions of severe respiratory depression between children with and without codeine use.

Codeine use among children in the United States has been declining^15^^–^^18^ following influential figures, such as the World Health Organization and the Centers for Disease Control, expressing concerns about the drug’s potential harm and questionable efficacy.^7^^,^^17^^,^^18^ In July 2017, the Japanese government also issued a position paper following these international guideline changes to announce the restriction of codeine use among children. Japan, however, did not take immediate action and set a 2-year grace period for the codeine restriction because (i) ultra-rapid metabolizers may be less prevalent in the Japanese population (in previous reports, the estimated prevalence of ultra-rapid metabolizers, who are at risk of fatal toxicity of codeine, has been estimated at 0.5% to 1% in Chinese and Japanese populations^5^^,^^19^); (ii) there had been no mortality case reports; and (iii) immediate restriction might cause confusion in clinical practice.

Previous reports have suggested that the estimated incidence of severe respiratory depression among Japanese children after any codeine use was less than 1%.^13^ In the present study, we confirmed that the proportion was much lower than previously reported: among 18,210 children who were prescribed codeine-containing antitussives, only seven (approximately 3.8 per 10,000 children) experienced severe respiratory depression. We also showed no significant association between codeine and the outcome of severe respiratory depression. Possible explanations for this insignificant association are (i) an underpowered sample size due to the rarity of severe respiratory depression after codeine use and (ii) physicians already being cautious about prescribing codeine-containing antitussives to children at risk of severe respiratory depression. Regarding the first explanation, we needed 69,013 cases to detect an odds ratio of 1.1, and we could only detect an odds ratio of 2.6 or more with the current cases.

Our findings have important clinical and health policy implications. Severe respiratory depression caused by codeine-containing drugs under current practice was quite rare and unobservable. Thus, there may be no substantial difference between the immediate and delayed restriction of codeine-containing antitussives in terms of preventing severe respiratory depression among children in Japan. Although our results did not eliminate the possibility of harmful effects of codeine-containing drugs, setting a 2-year grace period before restricting these drugs is unlikely to result in serious consequences.

This study had several limitations. First, we used administrative claims database that lacked detailed information on patient characteristics and outcomes. In particular, we were unable to obtain information on patients’ genetic phenotype, which defines drug metabolism, or the time from codeine intake to respiratory depression. Because diagnoses recorded in administrative claims databases are generally less accurate compared with those in planned prospective studies, miscoding of diagnoses may have affected our results if this miscoding was imbalanced between children with and without codeine use. Furthermore, coding inaccuracies may have resulted in under- or overestimation of respiratory depression cases. To adjust for the severity of the respiratory disease for which antitussives were prescribed, instead of information on clinical symptoms, we included type of medical institution and previous prescription of anti-asthma or COPD drugs as independent variables in the logistic regression analyses. Second, the data also lacked information on whether the patients took codeine-containing over-the-counter drugs. Third, the generalizability of the findings is limited because almost all of the patients were Japanese. The findings may not be applicable to non-Asian ethnic groups.

Nonetheless, the strength of our study is that we investigated the incidence of severe respiratory depression after the use of codeine-containing antitussives among children using a database that covers a very large number of individuals across all clinical settings.

In conclusion, severe respiratory depression among children after receiving codeine-containing antitussives was very rare, and an increase in adverse outcomes among users of codeine was undetectable, even with a large sample from the target population.
